# Relationship Between the Salivary Microbiome and Oral Malodor Metabolites in Older Thai Individuals with Periodontitis and the Cytotoxic Effects of Malodor Compounds on Human Oral Squamous Carcinoma (HSC-4) Cells

**DOI:** 10.3390/dj13010036

**Published:** 2025-01-16

**Authors:** Witsanu Srila, Kritsana Sripilai, Thunwa Binlateh, Peungchaleoy Thammanichanon, Watcharaphol Tiskratok, Parinya Noisa, Paiboon Jitprasertwong

**Affiliations:** 1 Division of Biology, Faculty of Science and Technology, Rajamangala University of Technology Thanyaburi, Pathum Thani 12110, Thailand; witsanu_s@rmutt.ac.th; 2 Laboratory of Cell-Based Assays and Innovations, School of Biotechnology, Institute of Agricultural Technology, Suranaree University of Technology, Nakhon Ratchasima 30000, Thailand; m6301408@g.sut.ac.th (K.S.); p.noisa@sut.ac.th (P.N.); 3 School of Pharmacy, Walailak University, Nakhon Si Thammarat 80160, Thailand; thunwa.bi@wu.ac.th; 4 Institute of Dentistry, Suranaree University of Technology, Nakhon Ratchasima 30000, Thailand; chaleoy@g.sut.ac.th (P.T.); watcharaphol@sut.ac.th (W.T.)

**Keywords:** halitosis, periodontitis, salivary microbiome, oral malodorous compound, salivary metabolomics, human oral squamous carcinoma cells, indole-induced apoptosis

## Abstract

**Background/Objectives**: Halitosis is primarily caused by the activity of oral microorganisms. In this study, we employed metagenomic sequencing and metabolomic approaches to investigate the differences in salivary microbiota and metabolite profiles between individuals with halitosis and periodontitis and healthy controls. Additionally, we expanded the study to examine how oral malodorous compounds interact with human oral squamous carcinoma (HSC-4) cells. **Methods**: Saliva samples were collected and analyzed using Ultra-High Performance Liquid Chromatography–Mass Spectrometry (UHPLC-MS) to identify metabolites. We then assessed the correlations between the microbiota and metabolites. Furthermore, the impact of oral malodorous substances on HSC-4 cells was investigated by evaluating apoptosis, antioxidant activity, and inflammatory properties. **Results:** The microbiota and metabolite profiles showed significant differences between the halitosis with periodontitis group and the periodontally healthy group. The halitosis with periodontitis group exhibited significantly higher relative abundances of eight genera: *Tannerella*, *Selenomonas*, *Bacteroides*, *Filifactor*, *Phocaeicola*, *Fretibacterium*, *Eubacterium saphenum*, and *Desulfobulbus*. In contrast, the periodontally healthy group showed significantly higher relative abundances of Family *XIII UCG-001*, *Haemophilus*, and *Streptobacillus*. Two metabolites, 2,3-dihydro-1H-indole and 10,11-dihydro-12R-hydroxy-leukotriene E4, were significantly higher in individuals with halitosis and periodontitis. In the treatment of HSC-4 cells with metabolites, dimethyl sulfide (DMS) did not show significant effects while indole appeared to induce cell death in HSC-4 cells by triggering apoptotic pathways. Additionally, both indole and DMS affected the inflammatory and antioxidant properties of HSC-4 cells. **Conclusions**: This study provides insights into the mechanisms of halitosis by exploring the correlations between microbiota and metabolite profiles. Furthermore, oral metabolites were shown to impact the cellular response of HSC-4 cells.

## 1. Introduction

Halitosis or oral malodor is considered by an unpleasant odor emitting from the oral cavity when talking or breathing, which can lead to social limitations and personal discomfort [1,2]. About 15–60% of people worldwide have halitosis [3]. The multifactorial etiology of halitosis involves intra-oral factors like inadequate oral hygiene, food impaction, unclean dentures, a coated tongue, and periodontal and gingival diseases, which account for 90% of cases, while systemic diseases like diabetes mellitus, gastrointestinal disorders, metabolic disorders, and respiratory diseases contribute to 10% of cases [4,5,6,7].

The oral cavity is a significant source of exhaled volatile organic compounds (VOCs) due to the oral bacteria metabolizing sugars and amino acids to produce various substances, including volatile sulfur compounds (VSCs), volatile fatty acids, and volatile nitrogen compounds [8,9,10]. Additionally, aromatic compounds, alcohols, aliphatic compounds aldehydes, and ketones also contribute to halitosis development [3]. VSCs, particularly hydrogen sulfide (H_2_S), methyl mercaptan (CH_3_SH), and dimethyl sulfide (DMS; CH_3_SCH_3_), are considered primary contributors to intra-oral halitosis [11,12,13,14]. In addition, other compounds, such as indole, skatole, putrescine, and cadaverine, may also contribute to the development of halitosis [15,16,17]. To assess halitosis, several methods are commonly used to measure VSCs, such as organoleptic measurements, halimeters, and gas chromatography (GC) [18,19,20]. However, these techniques only measure VSCs, but previous studies showed that other substances have an equally significant role in causing bad breath [3]. Recent research has focused on metabolic profiling, which can provide a better understanding of halitosis incidence and progression. Metabolic profiling has been applied to identify the biomarkers of asthma using liquid chromatography–mass spectrometry (LC-MS) [21] and different concentrations of metabolites were found in periodontally healthy and diseased patients using LC-MS and GC-MS [22,23,24].

*Actinomyces*, *Bacteroides*, *Dialister*, *Eubacterium*, *Fusobacterium*, *Leptotrichia*, *Peptostreptococcus*, *Porphyromonas*, *Prevotella*, *Selenomonas*, *Solobacterium*, *Tannerella*, and *Veillonella* are the most abundant genera in intra-oral halitosis patients [3]. Currently, advancements in next-generation sequencing (NGS) technology have made it possible to analyze the microbial communities related to halitosis including previously uncultured taxa [25,26]. As a result, we now have a better understanding of the changes in microecology that are associated with bad breath in the mouth. Several previous studies have demonstrated that intra-oral halitosis patients have higher bacterial diversity than healthy controls [27,28,29,30]. Halitosis is not only a result of microbial activity, but it also involves interactions between the pathogens and the host immune system [31,32]. In patients with periodontal disease, for instance, the chronic inflammation of the gingival tissues can provide an ideal environment for pathogenic bacteria to proliferate [29]. Furthermore, the host’s immune-inflammatory response can release additional substrates that may contribute to the production of VSCs. Metabolites from the microbiota can also influence host signaling pathways, exacerbating the inflammatory response and potentially leading to a positive feedback loop that promotes halitosis [32]. Understanding the pathogenesis of halitosis involves looking at the dynamic interactions between bacteria, metabolites, and host factors. In addition, identifying specific bacteria that drive the production of oral metabolites and understanding how these microorganisms interact within the oral microenvironment is essential to developing more effective treatments and prevention strategies for halitosis. Moreover, it has proven challenging to definitively determine whether microbes serve as mediators in the relationship between halitosis and periodontitis. Therefore, identifying the bacteria that affect the production of oral metabolites is essential to understanding intra-oral halitosis.

This work sought to clarify the variations in the microbiota and metabolite composition of the saliva from people with and without halitosis and periodontitis using metagenomics and LC-MS-based metabolite profiling. Saliva was used because it is accessible, easy to collect and store, and can be rapidly tested. It also contains a less complex VOC composition and fewer bioactive markers [21,33,34]. As a result, it is of interest to develop non-invasive diagnostic methods for assessing disease progression and treatment outcome using saliva samples. In addition, elevated levels of VSCs have been observed in both periodontitis and oral squamous cell carcinoma (OSCC) patients [35,36]. However, there is limited research exploring the relationship between these two conditions. Recent research has suggested a potential link between the accumulation of malodor substances, such as VOCs, and the development and progression of OSCC [35,37,38]. Previous studies have indicated that indole derivatives are able to inhibit the proliferation of leukemia cells [39] and breast cancer cells [40]. Additionally, DMS has been shown to induce apoptosis of leukemia cells through the activation of caspase-3 and the production of reactive oxygen species (ROS) [41]. However, the role of these substances in oral squamous carcinoma cells is limited and the precise mechanisms underlying this effect remain unclear. Therefore, we also aimed to investigate the potential influence of malodor substances on a human oral squamous carcinoma cell line (HSC-4).

## 2. Materials and Methods

### 2.1. Study Subjects and Clinical Examination

This study enrolled 22 participants from the Oral Health Center at the Suranaree University of Technology Hospital (Nakhon Ratchasima, Thailand) who underwent periodontal therapy between July 2023 and April 2024. A sample size calculation was conducted using G*Power version 3.1.9.7. Based on the results of Ye et al. [29], at least ten participants per group are needed to achieve 5% significance and 90% power. This study was approved by the Human Research Ethics Committee of the Suranaree University of Technology (EC-65-0081; date of approval: 3 November 2022) and all study procedures were performed in line with the principles of the Declaration of Helsinki. The subjects received complete information on the purposes and procedures of this study before they provided written informed consent. Two subject groups were included in the study: a periodontally healthy group (*n* = 10) and a periodontitis group (*n* = 12). The participants in the periodontally healthy group had clinically healthy gingiva, characterized by <10% bleeding on probing, probing depths ≤ 3 mm at all sites on an intact periodontium, and no clinical attachment loss or bone loss. The diagnosis of periodontitis in the periodontitis group was confirmed based on the following case definitions: (1) interdental clinical attachment loss detectable at ≥2 non-adjacent teeth, or (2) buccal or oral clinical attachment loss ≥ 3 mm with pocketing >3 mm that is detectable at ≥2 adjacent teeth. Experienced periodontists evaluated the full-mouth periodontal probing pocket depth (PPD), bleeding on probing (BOP) at six sites per tooth, clinical attachment level (CAL), and plaque index (PI) [42] scores, and then diagnosed the case as non-periodontitis, periodontitis stage I, II, III, or IV according to the classification criteria [43]. The exclusion criteria included (1) any uncontrolled systemic disease, i.e., diabetes mellitus, osteoporosis, or rheumatoid arthritis that may affect the patient’s periodontal status; (2) periodontal therapy within the previous 6 months; (3) pregnancy; (4) failure to sign an informed consent form; (5) possesses fewer than 20 teeth; and (6) past smoking history or current smoker.

### 2.2. Organoleptic Measurement

Each volunteer was instructed to keep their mouth closed for 1–2 min before sampling and to place a 4 cm section of the glass tube in their mouth, exhaling slowly through it. This process was repeated three times during each test. Each judge then evaluated the individuals’ mouth odor one at a time, with all three judges assessing the subjects. The organoleptic scores were independently recorded by each judge on an ordinal scale as follows: 0—no malodor; 1—slight malodor; 2—moderate oral malodor; 3—strong malodor (unacceptable intensity of mouth odor); and 4—very strong oral malodor.

### 2.3. Saliva Sample Collection, DNA Extraction, and Amplification of 16s rDNA

Samples were collected in a stimulated manner by asking the participants to chew paraffin wax (Xinsu Tech, Shenzhen, China) for 30 s, with the saliva ejected into a sterile 50 mL Falcon tube. The participants provided around 7.5–10 mL of saliva while under the supervision of a physician. There were no dietary restrictions for any of the subjects. The subjects were instructed to refrain from alcohol consumption for 12 h and not drink coffee or tea, smoke, or brush their teeth for 3 h before the sample collection. The saliva samples were promptly labeled and delivered to the laboratory, aliquoted (1 mL), and stored at −80 °C until further analysis. The untargeted metabolite profiles and microbiomes of the samples were analyzed using UHPLC-MS and metagenomic methods.

For microbial genomic DNA isolation, the saliva samples were vortexed, centrifuged, and the DNA was then extracted using a Nextractor NX-Jr machine and an NX-Jr Genomic DNA Kit (Genolution, Gangseo-gu, Seoul, Republic of Korea) in accordance with the manufacturer’s manual. The genomic DNA (gDNA) concentration and purity were determined using a Nano-Drop 2000 (Thermo Scientific, Waltham, MA, USA). The quality of the gDNA was evaluated using 1% agarose gel electrophoresis. The V3/V4 regions of the 16S rDNA of the microbial gDNA samples were amplified using PCR and specific primers (341F: 5′-CCTACGGGNGGCWGCAG-3′; 805R: 5′-GACTACHVGGGTATCTAATCC-3′) according to the standard protocols provided by U2Bio (Songpa-gu, Seoul, Republic of Korea).

### 2.4. Metagenomics and Data Processing

The amplicons were pooled in an equimolar ratio and the pooled libraries were sequenced using the Illumina MiSeq platform according to the standard protocols provided by U2Bio (Songpa-gu, Seoul, Republic of Korea). To create the datasets, the adapter sequences were trimmed and the primer sequences were removed from the raw sequencing reads using cutadapt V.1.15 [44]. The raw sequencing reads were then quality filtered and merged, and chimeras were removed using the DADA2 pipeline [45]. Taxa were clustered through an amplicon sequence variant (ASV) analysis using the SILVA v.138.1 database. The microbiome profiles were created and all analyses were conducted using the phyloseq package in R version 4.1.0 [46]. The analysis was performed at the phylum to genus level.

### 2.5. UHPLC-MS Analysis

Saliva samples from the halitosis with periodontitis and periodontally healthy groups were thawed at room temperature. Equal volumes (1 mL) of the saliva samples from the two groups were prepared according to a previously described method [47]. The analyses were conducted using a Dionex Ultimate 3000 UHPLC system (Dionex, Sunnyvale, CA, USA) connected to an electrospray ionization (ESI) tandem mass spectrometer (micrOTOF-Q II) (Bruker, Bremen, Germany). An injection volume of 8 μL was used for all samples. Separation was performed on a Zorbax SB-C18 column (250 mm × 4.6 mm × 3.5 μm; Agilent Technologies, Santa Clara, CA, USA), maintained at 30 °C, with a flow rate of 0.5 mL/min. The mobile phase consisted of deionized water with 0.1% formic acid as solvent A, and methanol with 0.1% formic acid as solvent B. The gradient elution was carried out using the following solvent gradient: 5% B for the first 5 min, increasing to 30% B at 30 min, then to 95% B at 50 min and maintained until 58 min. Afterward, the gradient was reduced to 5% B over 2 min and held until the end of the run at 65 min. The eluted compounds were ionized by the ESI source and detected in mass scanning mode within the range of 50 *m*/*z* to 1500 *m*/*z* under positive ion polarity. For accurate mass calibration, a 10 mM sodium formate solution was used as the external standard, injected into the ESI source before each sampling via a syringe pump at a flow rate of 60 µL/min. The pressure of the nebulizer gas (N_2_) was 2 Bar, the drying gas flow rate was 8 L/min, the dry heater temperature was 220 °C, and the capillary voltage was 4.5 kV. The LC-QTOF data were collected and processed using Compass 1.3 software (Bruker, Bremen, Germany). The fragmentation patterns of the substances were predicted from databases because reference standards were not available. The data filtering and alignment process was conducted using the following parameters: molecular features with an intensity greater than 10,000 cps and a mass alignment window of 10 ppm. The metabolites were tentatively annotated and characterized using the Human Metabolome Database (HMDB) [48].

### 2.6. Cell Culture

Human squamous carcinoma (HSC-4) cells (JCRB0624) were sourced from the Japanese Collection of Research Bioresources (JCRB) Cell Bank. The cells were cultured in complete medium consisting of HyClone Dulbecco’s Modified Eagle Medium (DMEM) with high glucose and L-glutamine (Cat. #SH30243.02, Cytiva, Marlborough, MA, USA), supplemented with 10% fetal bovine serum (FBS; Cytiva, Marlborough, MA, USA) and 1× penicillin–streptomycin (Thermo Fisher Scientific, Waltham, MA, USA). They were grown in 6-well plates or T-flasks (Corning, Oneonta, NY, USA) and incubated at 37°C with 5% CO_2_ and humidified air. The cells were passaged when they reached 80–90% confluency using 1× TrypLE™ Express Enzyme (Thermo Fisher Scientific, Waltham, MA, USA).

### 2.7. Treatment and Cell Viability Assay

Cell viability was assessed using a colorimetric methyl-thiazolyl tetrazolium (MTT) assay. HSC-4 cells were plated in 96-well plates at a density of 1.0 × 10^4^ cells per well in complete medium and incubated for 24 h. Once the cells reached 80% confluency, they were treated with various reagents in DMEM at different concentrations or with the vehicle (0.1% DMSO) for 24–48 h. Following treatment, the cells were washed with PBS, and 0.5 mg/mL of MTT solution was added to each well and incubated at 37 °C for 3 h. After incubation, the MTT solution was removed, and DMSO (100 μL) was added to dissolve the formazan crystals. The absorbance for each treatment was then measured at 570 nm using a microplate reader (BMG Labtech, Ortenberg, Germany).

Indole (C_8_H_7_N; Tokyo Chemical Industry, Tokyo, Japan) and DMS (Sigma-Aldrich, St. Louis, MO, USA) were dissolved in dimethyl sulfoxide (DMSO) and phosphate-buffered saline (PBS), respectively. Hydrogen peroxide (H_2_O_2_) and lipopolysaccharides (LPSs; Sigma-Aldrich, St. Louis, MO, USA) in DMEM were used as the controls.

### 2.8. Total RNA Extraction and RT-qPCR

Total RNA was isolated using a NucleoSpin RNA Plus kit (Macherey-Nagel, Dueren, Germany) according to the manufacturer’s guidelines, and 800 ng of total RNA was converted into cDNA using a ReverTra Ace^®^qPCR RT Master Mix with gDNA Remover (Toyobo, Osaka, Japan). For the qPCRs, one microliter of the diluted cDNA was used as a template in 10 µL reactions using the 2× qPCRBIO SyGreen Lo-Rox Mix (PCR Biosystems, London, UK). The reactions were performed with specific primers (Table S1) in quadruplicates using a QuantStudio^TM^ 3 Real-Time PCR System (Thermo Fisher Scientific, Waltham, MA, USA), as previously described [49]. The relative gene expression was normalized to GAPDH (glyceraldeyde-3-phoshate dehydrogenase) expression for the fold change calculations.

### 2.9. Enzyme-Linked Immunosorbent Assay (ELISA)

ELISA experiments for IL-6 (Human IL-6 ELISA Kit, Cat. #ab178013) and IFN-γ (Human IFN gamma ELISA Kit, Cat. #ab174443) were performed according to the manufacturer’s protocols (Abcam, Cambridge, MA, USA).

### 2.10. Statistical Analysis

All data are expressed as the mean ± standard deviation. The statistical procedures used in this study included *t*-tests for the demographic and clinical characteristics of the patients. Bray–Curtis distances were used to measure the dissimilarity or difference between two samples based on their species or taxon composition. Statistical analyses were conducted using one-way ANOVA, followed by Dunnett’s multiple comparisons test for cell viability, ELISA, and qPCR results, all performed with GraphPad Prism 8 software. A significance level of *p* < 0.05 was considered statistically significant. The symbols “a, b, and c” represent statistically significant differences at *p* < 0.001, 0.01, and 0.05, respectively. Comparisons between the halitosis with periodontitis group and the periodontally healthy group were performed using a Student’s *t*-test. The differences in salivary microbiota compositions were analyzed using the Mann–Whitney U test.

## 3. Results

### 3.1. Clinical Characteristics and Periodontal Parameters

The classification of the study groups was based on the details laid out in Section 2. The organoleptic test served as a standard for assessing oral halitosis. The clinical characteristics and periodontal parameters of the participants are shown in Table 1. The organoleptic scores and all the periodontal parameters, including PPD, BOP, and CAL, were significantly higher in the halitosis with periodontitis group than the periodontally healthy group. No significant differences in plaque index scores were observed between the groups.

### 3.2. Salivary Microbiome

**Figure 1:**
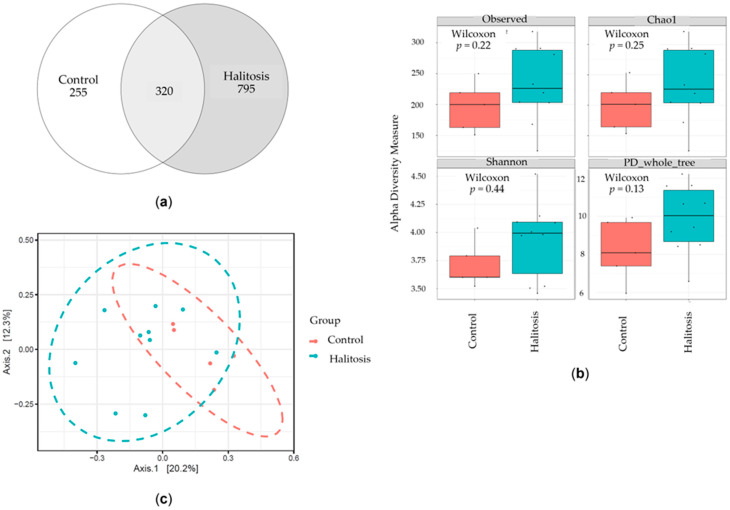
Comparison of the microbial richness and diversity of saliva samples from the halitosis with periodontitis and the periodontally healthy groups. (**a**) The Venn diagram illustrates the number of amplicon sequence variants (ASVs) that are shared and distinct between the groups, providing insight into the similarity and overlap of the ASVs between the groups. (**b**) Comparison of the alpha diversity indices between the halitosis with periodontitis and periodontally healthy groups. (**c**) The PCoA at the ASV level was based on the Bray–Curtis distances between the salivary microbial communities in the groups (*p* < 0.05).

The periodontal microbial composition of the saliva of the ten patients in the halitosis with periodontitis group was compared to that of five individuals without periodontitis (periodontally healthy group). The overlapping and distinct communities between the groups are illustrated by the Venn diagram in Figure 1a. The overall number of amplicon sequence variants (ASVs) was 1370. Among these, 320 ASVs were common to both groups, while 255 ASVs were exclusive to the periodontally healthy group and 795 ASVs were specific to the halitosis group. These findings indicate an increase in oral microbial diversity in the halitosis with periodontitis group.

The alpha diversity, which measures richness, dominance, and evenness, was calculated within a single population to compare the diversity of the saliva microbiota between the two groups. The observed species, Chao1 index, phylogenetic diversity (PD whole tree), and Shannon index scores were used to describe the alpha diversity (Figure 1b). The mean index value of the halitosis with periodontitis group was slightly higher than that of the control group. Nevertheless, there were no statistically significant differences in the abundance and diversity of the microbial communities between the two groups (*p* > 0.05, Mann–Whitney U test). In addition to the alpha diversity evaluation, principal coordinates analysis (PCoA) was also used to compare the overall microbial communities (beta diversity) between the groups. The Bray–Curtis distance exhibited a difference in the PCoA plot between the halitosis with periodontitis and periodontally healthy groups (*p* < 0.05), even though some samples overlapped (Figure 1c).

**Figure 2:**
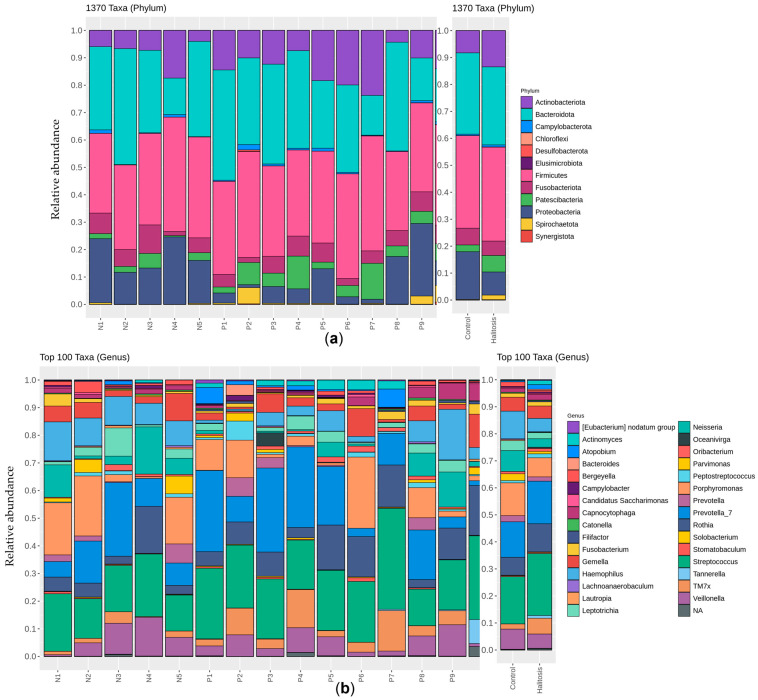
The composition of the salivary microbial communities in the groups at the phylum (total taxa; (**a**)) and genus (top 100 taxa; (**b**)) levels. The 16S rRNA gene sequences were input into DADA2 for quality filtering, clustered into ASVs, and the taxa were assigned based on the SILVA v.138.1 database.

Subsequently, we categorized the taxa that contributed to the overall differences in the communities between the two groups. The microbial community profiling of each sample at the phylum level is displayed in Figure 2a. The microbial composition of both groups showed that *Actinobacteriota*, *Bacteroidota*, *Campylobacterota*, *Firmicutes*, *Fusobacteriota*, and *Spirochaetota* were abundant in all samples, despite the presence of halitosis with periodontitis. The abundances of *Desulfobacterota* (*p* = 0.012), *Patescibacteria* (*p* = 0.04), and *Synergistota* (*p* = 0.012) were notably higher in the halitosis with periodontitis group than the periodontally healthy group (Figure S1). In contrast, the abundance of *Proteobacteria* (*p* = 0.055) was lower in the halitosis with periodontitis group than the periodontally healthy group. However, the microbial pattern of some individuals without periodontitis was comparable to that of the periodontitis patients. The halitosis with periodontitis group’s microbial patterns differed from those of the periodontally healthy group at the genus level (Figure 2b and Figure S2). The most commonly found genera in both groups were *Actinomyces*, *Bergeyella*, *Campylobacter*, *Capnocytophaga*, *Fusobacterium*, *Gemella*, *Leptotrichia*, *Neisseria*, *Oribacterium*, *Parvimonas*, *Peptostreptococcus*, *Porphyromonas*, *Prevotella*, *Prevotella 7*, *Rothia*, *Streptococcus*, *Saccharimonadaceae*, *TM7x*, *Treponema*, and *Veillonella*, which had mean relative abundances greater than 1%. Moreover, we also found that some genera in the halitosis with periodontitis group had higher mean relative abundances (*Eubacterium nodatum*, *E. brachy*, *Actinobacillus*, *Actinomyces*, *Campylobacter*, *Candidatus Saccharimonas*, *Cardiobacterium*, *Centipeda*, *Corynebacterium*, *Dialister*, *F0058*, *F0332*, *Fusobacterium*, *Kingella*, *Lachnoanaerobaculum*, *Megasphaera*, *Mycoplasma*, *Oribacterium*, *Peptococcus*, *Prevotella 7*, *Rothia*, *Solobacterium*, *Stomatobaculum*, *Streptococcus*, and *TM7x*) than the periodontally healthy group (Figure S2). In contrast, some genera in the periodontally healthy group had higher mean relative abundances (*E. yurii*, *Abiotrophia*, *Bergeyella*, *Catonella*, *Eikenella*, *Gemella*, *Granulicatella*, *Johnsonella*, *Lautropia*, *Leptotrichia*, *Neisseria*, *Parvimonas*, *Porphyromonas*, *Prevotella*, and *Veillonella*) than the halitosis with periodontitis group. The relative abundances of *E. saphenum*, *Bacteroides*, *Desulfobulbus*, *Filifactor*, *Fretibacterium*, *Phocaeicola*, *Selenomonas*, and *Tannerella* were considerably higher in the halitosis with periodontitis group, whereas the relative abundances of *Family XIII UCG-001*, *Haemophilus*, and *Streptobacillus* were significantly higher in the periodontally healthy group (*p* ˂ 0.05; Figure S2).

**Figure 3:**
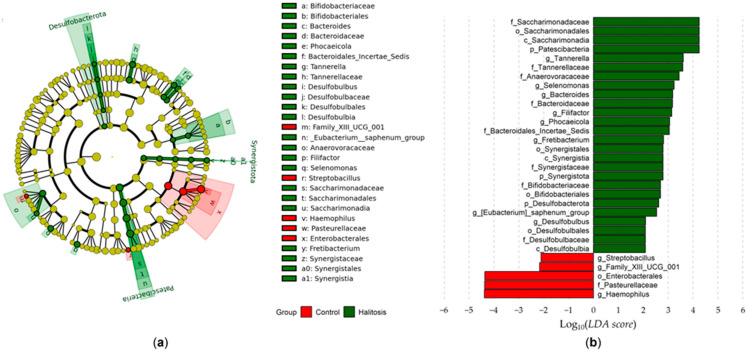
The differentiation of bacteria taxa between the halitosis with periodontitis and periodontally healthy groups was performed using LEfSe analysis. The bacteria in the saliva samples with an LDA score > 2 are displayed. (**a**) The cladogram generated from the LEfSe analysis revealed distinct microbial clades present in both groups. The colored regions/branches represent discrepancies in the bacterial population structure between the two groups. The green sectors represent clades that are more abundant in the halitosis with periodontitis group compared to the periodontally healthy group, whereas the red sector represents clades that are more abundant in the control group compared to the halitosis with periodontitis group. (**b**) The LDA scores indicate substantial differences in bacterial composition at the genus level between the halitosis with periodontitis and periodontally healthy groups.

We also applied Linear discriminant analysis Effect Size (LEfSe) analysis to determine the features that most likely explain the differences between the two groups based on relative abundances. A cladogram using the LEfSe results was used to display the different bacterial compositional structures (Figure 3a). Figure 3b shows the differences based on the linear discriminant analysis (LDA) score (LDA score > 2). The analytical results showed that the halitosis with periodontitis and periodontally healthy groups had significantly different bacterial compositions at the genus level. The halitosis with periodontitis group had significantly higher relative abundances of twenty-six taxa compared to the periodontally healthy group, while the periodontally healthy group had significantly greater relative abundances of five taxa compared to the halitosis with periodontitis group. *Patescibacteria*, *Saccharimonadia*, *Saccharimonadales*, and *Saccharimonadaceae* showed the highest LDA score among all the microbes associated with halitosis. Five bacterial genera, *Tannerella*, *Selenomonas*, *Bacteroides*, *Filifactor*, and *Phocaeicola*, were higher in the halitosis with periodontitis group than in the periodontally healthy group, and these bacteria were significantly associated with halitosis.

### 3.3. Metabolite Profiling

The saliva samples were analyzed using UHPLC-MS. Twenty-six metabolites exhibited significant differences in concentration between the halitosis with periodontitis and periodontally healthy groups. The sum of these metabolites is tentatively predicted in Table 2, and their annotation is tentatively provided in Table S2. A total of 19 out of the 26 metabolites were able to be tentatively characterized and annotated. Eleven significant compounds exhibited a higher abundance in the group with halitosis and periodontitis, whereas five compounds had a lower abundance in the periodontally healthy group. The concentration of six compounds was notably higher in the group of individuals with halitosis and periodontitis. However, four compounds were only detected in the periodontally healthy group. Two *m*/*z* values, 120.0821 and 458.2525, were detected at noticeably greater levels in the halitosis group, as indicated in Table S2. The *m*/*z* values of 120.0821 and 458.2525 were identified as 2,3-dihydro-1H-indole (C_8_H_9_N) and 10,11-dihydro-12R-hydroxy-leukotriene E4 (C_23_H_39_NO_6_S), respectively.

**Figure 4:**
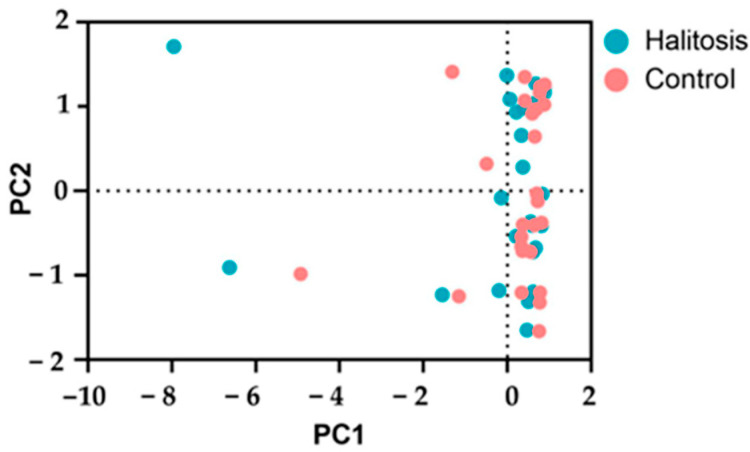
The principal component analysis (PCA) of the UHPLC-MS metabolic profiles of the saliva samples from the halitosis with periodontitis and periodontally healthy control groups.

A principal component analysis (PCA) was initially used to further examine the variations in metabolic profiles between the periodontally healthy and halitosis groups. However, the PCA was unable to distinguish between the two groups (Figure 4).

### 3.4. Effect of Malodor Substances on HSC-4 Cell Lines

**Figure 5:**
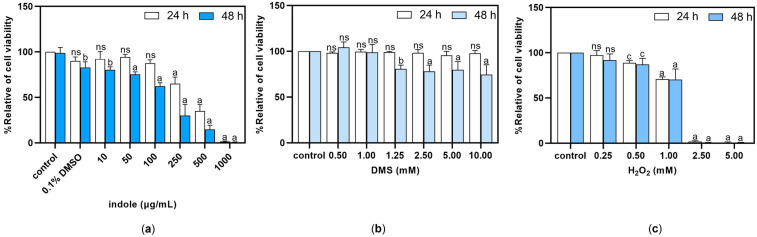
Viability of HSC-4 cells after treatment with indole (**a**), DMS (**b**), and H_2_O_2_ (**c**). The cells were incubated with the vehicle (0.1% DMSO or PBS) or various concentrations of indole, DMS, or H_2_O_2_ for 24 or 48 h, and cell viability was assessed using the MTT assay. The data are presented as the mean ± SD (*n* = 3). Statistical significance was analyzed using one-way ANOVA and followed by Dunnett’s multiple comparisons test: ^c^ *p* < 0.05, ^b^ *p* < 0.01, ^a^ *p* <0.001, and ^ns^ not significant.

We conducted a study to examine the effects of indole and DMS on the viability of HSC-4 cells. In the halitosis with periodontal group, there were high levels of indole derivatives, followed by 10,11-dihydro-12R-hydroxy-leukotriene E4 and DMS, which is considered a significant contributor to intra-oral halitosis. The cells were exposed to different doses of these substances (Figure 5a–c) and to H_2_O_2_, which was utilized as a positive control in this experiment. Subsequently, the assessment of cell viability was conducted using the MTT assay. In the positive control experiment, the growth of the HSC-4 cells was considerably decreased by the H_2_O_2_ treatments in a dose-dependent manner relative to the untreated control cells (Ctrl). Exposing the HSC-4 cells to a concentration of more than 100 μg/mL of indole for 24 h resulted in a significant decrease in cell viability. Similarly, treating the HSC-4 cells with different concentrations of indole for 48 h hindered cell proliferation in a dose-dependent fashion, relative to the control group. When the HSC-4 cells were treated with DMS, their viability remained unaffected after 24 h. However, a concentration of more than 1 mM DMS decreased cell viability after 48 h of treatment.

**Figure 6:**
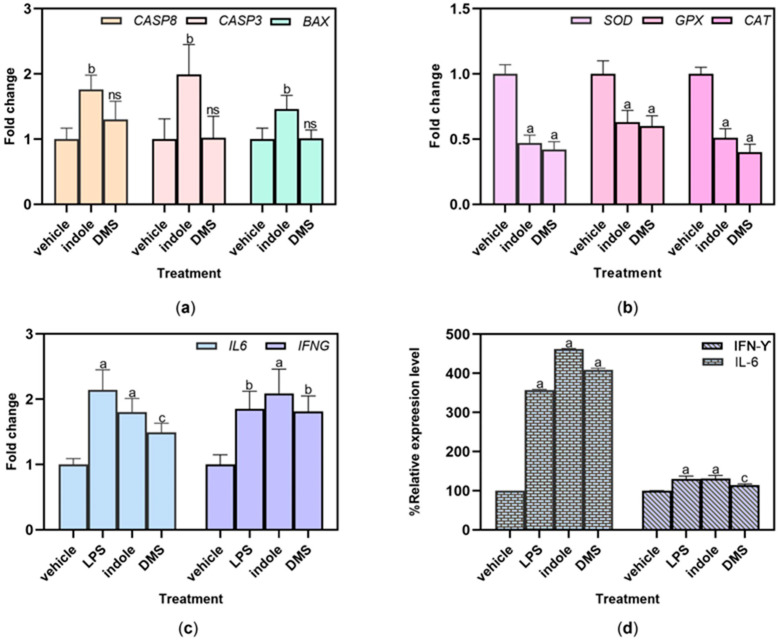
The effect of indole and DMS on apoptosis and antioxidant and inflammatory properties of HSC-4 cells. The mRNA expression levels of apoptosis- (**a**), antioxidant- (**b**), and inflammation-related (**c**) genes were quantified using RT-qPCR, and the GAPDH gene was used as the reference gene. (**d**) The relative expressions of IFN-γ and IL-6 were assayed using ELISA. The expression levels of all genes and secreted cytokines of HSC-4 cells after treatment with 100 μg/mL indole and 5 mM DMS were compared to those of untreated control cells. The values are presented as the mean ± SD (*n* = 3). ^c^ *p* < 0.05, ^b^ *p* < 0.01, ^a^ *p* < 0.001, and ^ns^ not significant versus vehicle.

The HSC-4 cells were exposed to 100 μg/mL indole and 5 mM DMS to study the mechanism underlying the apoptosis caused by both substances. RT-qPCR was used to assess the expression of genes associated with apoptosis, such as CASP3 (caspase-3), CASP8 (caspase-8), and BAX. These three pro-apoptotic genes were considerably elevated by the indole treatment (Figure 6a). In contrast, the DMS treatment had no effect on the expression of these three genes.

We assessed the influences of indole and DMS on the expression of genes encoding the antioxidant enzymes including superoxide dismutase (SOD), glutathione peroxidase (GPX), and catalase (CAT). The expression levels of these genes were significantly decreased after treatment with 100 μg/mL indole and 5 mM DMS compared to the untreated cells (Figure 6b).

The inflammatory gene markers IL6 and IFNG were selected to study the expression of inflammatory activation after the indole and DMS treatments. Compared to the vehicles, the indole and DMS treatments substantially upregulated the expression of both inflammatory genes in HSC-4 cells (Figure 6c). We further investigated the secretion of inflammatory-related cytokines, such as IFN-γ and IL-6, using ELISA. In HSC-4 cells, the indole and DMS treatments dramatically raised the relative expression levels of the IL-6 and IFN-γ cytokines (Figure 6d).

## 4. Discussion

The oral cavity is a crucial and intricate environment within the body, hosting a diverse microbial community that may influence oral health or disease. Previous research has indicated that microbiome variations are frequently observed in diseased regions [3,29,34]. The intricate interplay between the oral microbiome and host immune responses has become an attractive area of study [50]. The present study explored variations in the salivary microbiome associated with halitosis in individuals with periodontitis and compared these with the microbiome and metabolites found in periodontally healthy Thai individuals. The goal of this research was to support the development of non-invasive, cost-effective diagnostic techniques that use saliva to detect periodontal diseases.

Next-generation sequencing methods have been applied to assess differences in microbial composition in periodontitis patients. Recent research on the oral microbiome has shown that the V3/V4 regions are appropriate for both capturing the richness of the overall community and performing taxonomic classification at the bacterial species level. Moreover, using longer amplicons encompassing the V3–V4 region yield can increase the detection of bacterial populations and beta diversity. The V3–V4 hypervariable region primer pairs are more resilient to spiking with human DNA and have greater capture abilities than primer pairs spanning the V1–V3 region [51]. Here, we utilized the Illumina MiSeq platform system with the V3–V4 hypervariable regions, which provides the advantages of high-quality sequencing, easy data analysis, and low error rates and costs, to sequence the microbial genome for the periodontitis and healthy groups.

In this study, periodontally healthy patients served as controls to account for the influence of periodontal factors. Additionally, there were no notable differences in plaque index scores between the groups. This implies that the composition of bacteria, rather than the quantity of plaque, could be the underlying factor in oral halitosis associated with periodontitis. Thus, analyzing the bacterial composition of the saliva microbiome could offer valuable insights into the major organisms associated with the development of oral halitosis along with periodontitis. The ecological diversity of the microbiome, as measured by the Shannon index, has been proposed as a predictor for periodontal status [1]. However, no statistically significant differences were observed in the abundance and diversity of the microbial communities in the two groups in this study. Some studies reported that the observed operational taxonomic units (OTUs) and Chao1 [28], Shannon, and Simpson diversity indices of healthy and halitosis saliva samples showed no significant differences. Another study demonstrated that the observed species and Chao1 diversity indices were markedly higher in the halitosis with periodontitis group than in the healthy group [27]. There were significant discrepancies in beta diversity between the two groups, in accordance with a previous study [27]. This finding indicates that the diversity of microbial communities may be a potential cause of halitosis.

Bacteria of the genera *Actinomyces*, *Bergeyella*, *Campylobacter*, *Capnocytophaga*, *Fusobacterium*, *Gemella*, *Leptotrichia*, *Neisseria*, *Oribacterium*, *Parvimonas*, *Peptostreptococcus*, *Porphyromonas*, *Prevotella*, *Prevotella 7*, *Rothia*, *Streptococcus*, *Saccharimonadaceae*, *TM7x*, *Treponema*, and *Veillonella* were found in both the halitosis with periodontitis and the periodontally healthy groups. These bacteria are not commonly linked to periodontitis and microorganisms found in saliva. The most prevalent genera did agree with the findings of previous research [29,52,53].

Using LEfSe analysis, we aimed to identify bacterial species that could serve as biomarkers for periodontitis associated with halitosis. We found that the bacterial composition at the genus level in the halitosis with periodontitis group was significantly different from that in the periodontally healthy group. *Tannerella*, *Selenomonas*, *Bacteroides*, *Filifactor*, and *Phocaeicola* were among the bacteria species that were most strongly associated with halitosis with periodontitis and could be used as biomarkers for this condition. The bacteria that were more prevalent in the halitosis with periodontitis group may be directly linked to the synthesis of metabolites that contribute to the development of halitosis. Chen et al. [52] revealed that the following genera were correlated with subgingival plaque in individuals with periodontitis: *Filifactor*, *Tannerella*, *Eubacterium*, *Desulfobulbus*, *Phocaeicola*, and *Mycoplasma*. *Granulicatella*, *Eubacterium*, and *Prevotella* are prevalent in the oral microbiota and were strongly positively correlated with the generation of VSCs, a major cause of halitosis. Ye et al. [29] reported that *Solobacterium*, *E. nodatum*, and *Parvimonas* are significantly related to methyl mercaptan production, while *Leptotrichia*, *Veillonella*, *E. nodatum*, and *Candidatus Saccharimonas* are significantly associated with DMS production. *Bacteroides*, *Selenomonas*, and *Fretibacterium* have recently been found to be positively associated with periodontal pathogens [54,55]. A previous investigation also indicated that the microorganisms most strongly linked to bad breath are *Atopobium parvulum*, *Dialister*, *E. sulci*, *TM7*, *Solobacterium*, and *Streptococcus* [29]. *Bergeyella*, *Veillonella*, *Haemophilus*, and *Rothia* were not associated with halitosis [29,52,56]. We observed that certain genera exhibited correlations with both disease and health, indicating opposing periodontal health effects within the same genera of bacteria.

The findings from a previous report [5] support the idea that certain microorganisms in saliva samples differ from those in tongue samples. These differences may be attributed to the distinct microbial compositions of the tongue coating, tooth-associated plaque, and saliva samples, as well as the uncertain impact of halitosis on saliva microbiota patterns. Furthermore, there is limited research on the microbiota of saliva [27].

Our investigation identified six metabolites with varying abundances in the halitosis patients with periodontitis but were lacking in the periodontally healthy group, and eleven metabolites that were higher in the halitosis patients with periodontitis compared to the periodontally healthy group among the 26 assessed molecular features (*m/z* values). These metabolites exhibited a correlation with the prevalence of different periodontal bacteria. The variations in the PCA results within the groups did not reveal a substantial clustering of the data. This might be because the difference within one group was not low enough compared to the variation between the groups. The identified metabolites in this study were distinct from those reported previously by Bregy et al. [47]. The use of different assessment procedures, criteria for admission, subject races, sequencing segments, and databases could be potential causes of these distinct results [29]. Therefore, more research is required to confirm these results. In addition, there are not many studies on saliva metabolites that used UHPLC-MS.

To study the correlation between the salivary microbiome and metabolite profile, we investigated the interactions between the identified compounds and microbiota. This study found that the halitosis with periodontitis group had significantly greater levels of eight genera, namely *Tannerella*, *Selenomonas*, *Bacteroides*, *Filifactor*, *Phocaeicola*, *Fretibacterium*, *Eubacterium saphenum*, and *Desulfobulbus*, compared to the periodontally healthy group (Figure 2b). These genera are frequently identified in people with halitosis [3,23,52,57]. These genera exhibited a positive correlation with 11 upregulated metabolites, as shown in Table 2. These findings indicate that metabolites may be linked to the development of halitosis.

Two metabolites, 2,3-dihydro-1H-indole and 10,11-dihydro-12R-hydroxy-leukotriene E4, were identified in our study as the most significantly elevated in periodontitis-associated oral malodor. These metabolites could potentially serve as indicators for detecting halitosis in people with periodontitis. 2,3-dihydro-1H-indole (indoline) is an indole. Other indoles, such as indole and skatole, are believed to be responsible for bad breath. These compounds are produced by certain types of bacteria found in the oral cavity, specifically anaerobic Gram-negative bacteria like *Porphyromonas intermedia*, *Fusobacterium nucleatum*, *Porphyromonas gingivalis*, and *Bacteroides* as well as the Gram-positive *Streptococcus milleri* [15,58]. This aligns with our study, which identified the presence of *Bacteroides* and *Fusobacterium* in the group of individuals with halitosis and periodontitis. This substance was also found in the gas chromatography–mass spectrometry (GC-MS) analysis, as shown in Figure S3. 10,11-Dihydro-12R-hydroxy-leukotriene E4 is classified as a hydroxyeicosatrienoic acid and is produced when leukotriene B4 is metabolized by human keratinocytes. Hydroxyeicosatrienoic acids are potent mediators of inflammation and seem to play a role in the characteristic symptoms of allergic rhinitis [59]. Bregy et al. [47] detected long-chain fatty acids, including hydroxydodecapentaenoic acid, in periodontitis patients. Barnes et al. [22] identified 40 known and 32 unknown metabolites that were higher in periodontitis patients than in the healthy control group. They found several fatty acid derivatives, such as dihomo-linolenate, arachidonate, docosapentaenoate, and docosahexaenoate, that are precursors to inflammatory substances, such as eicosanoids. These metabolites can be produced by the Gram-negative bacteria inhabiting periodontal pockets and the dorsum of the tongue after breaking down food particles, cells, saliva, and blood components [47,60].

The oral microbiota from periodontitis patients (in which *Porphyromonas* was the most abundant genus) was found to facilitate the onset of oral squamous cell carcinoma in a previous study. This was accompanied by changes in the oral bacterial community and the tumor immune microenvironment [36]. *Porphyromonas* also produces indole [58], which is considered to be a contributing factor to the development of oral halitosis. Some previous study reported that indole derivatives could effectively inhibit myeloid leukemia cell proliferation [39] and breast cancer cell growth by inducing the apoptosis pathway [40]. DMS has a greater number of sulfur atoms and could more effectively decrease human leukemia cell viability and induce apoptosis in human leukemia cells by inducing apoptosis through the activation of caspase-3 and the production of ROS [41]. Moreover, the levels of these VSCs are higher in both periodontitis and OSCC patients [35,36]. Therefore, we questioned whether indole and DMS affect cell viability and apoptosis-, antioxidant-, and inflammatory-related genes in HSC-4 cells.

We found that treatment with indole at concentrations of 250–1000 μg/mL for 24 h or at 50–1000 μg/mL for 48 h significantly inhibited the growth of HSC-4 cells compared to the untreated control cells. Noroozi et al. [39] found that newly developed indole derivatives effectively decreased the growth of the acute promyelocytic leukemia NB4 cell line. Furthermore, different derivatives exhibited varying levels of effectiveness in reducing cell viability. Treatment with DMS at 1.25–10.0 mM for 48 h significantly decreased the cell viability of the HSC-4 cells. Zhang et al. [41] demonstrated that treatment with 5 and 20 μM DMS for 24–48 h resulted in a slight decrease in the viability of Jurkat and HL-60 cells. The variation in dosage of DMS and indole among different cell lines could trigger their differentiation into distinct lineages. The results demonstrated that DMS and indole exhibit cytotoxic effects on HSC-4 cells in a dose- and time-dependent manner, displaying a degree of specificity toward these cells.

In order to clarify the apoptosis mechanism induced by indole and DMS, the apoptosis-related genes *CASP3*, *CASP8*, and *BAX* were examined. These pro-apoptotic genes exhibited a significant increase in expression after the indole treatment, while no expression was observed after the DMS treatment. These results indicated that indole inhibits the growth of HSC-4 cells. These findings are in line with earlier studies showing that DMS had no discernible effect on the apoptosis of Jurkat and HL-60 cells [41] and that indole derivatives (C19H15F3N2O and I3C) accelerated the death of NB4 and H1299 cells [39,61]. According to our findings, indole may cause HSC-4 cells to undergo apoptosis by activating the intrinsic apoptosis pathways regulated by the BCL-2 family (*BAX*) and the extrinsic pathways of apoptosis regulated by the executioner caspase enzyme (caspase-3 and caspase-8).

The treatments with indole and DMS significantly decreased the expression of all the tested antioxidant enzyme-related genes (*GPX*, *SOD*, and *CAT*) in HSC-4 cells compared to the untreated cells. According to previous research, rat liver catalase activity is reduced by indole acetic acid treatment [62]. A previous report suggested that DMS functions as an antioxidant in cells [63]. Intracellular ROS levels can harm DNA, proteins, and organelles, which can result in apoptosis. Our findings demonstrated that DMS and indole dramatically reduced the expression of antioxidant genes in the cells. Based on these results, indole may induce apoptosis in HSC-4 cells through ROS-dependent pathways. Additionally, our findings showed that treatment with indole or DMS significantly increased the expression of inflammation-related genes and cytokines. These results suggest that both substances may trigger HSC-4 cells to release inflammatory cytokines. Indole derivatives are known to activate plant immunity against pathogens and herbivorous insects, and they may also influence the immune system in humans [58,64]. Taken together, these findings suggest that indole and DMS could both act as pro-inflammatory agents and induce cell death via oxidative stress in cancer cells. This immune system modulation might present opportunities for future research targeting specific cellular pathways involved in periodontitis-associated cancer progression. However, the sample size in this study was relatively small, which may limit the generalizability of the findings. Larger and more diverse cohorts are needed to confirm the results and assess whether the identified microbial patterns are consistent across different populations and geographic regions.

## 5. Conclusions

This study explored the microbial community in halitosis with periodontitis patients. The halitosis with periodontitis group showed a higher microbial diversity compared to the periodontally healthy group and several bacteria were significantly associated with halitosis development. This study also demonstrated correlations between specific microbiota and metabolites and this condition. A better understanding of how the oral microbiome and malodor metabolites influence oral health, particularly in patients with periodontitis, may lead to novel diagnostic and therapeutic strategies for managing oral malodor and periodontal diseases. Furthermore, we extended this study to the investigation of the effect of oral malodorous substances on HSC-4 cells. Indole may induce cell death in HSC-4 cells by activating the intrinsic apoptosis and extrinsic apoptosis pathways, while DMS does not. Indole and DMS could also affect the antioxidant and inflammatory properties of HSC-4 cells. The role of oral malodor compounds could potentially contribute to the development of preventive or adjunctive therapies for oral cancer and periodontal diseases. This study underscores the importance of integrating microbiome research into clinical practice to improve patient outcomes in oral health.

## Figures and Tables

**Table 1:** Demographic and clinical characteristics of patients.

Variable	Periodontally Healthy Controls (*n* = 10)	Periodontitis Patients (*n* = 12)
Age (years)	22.34 ± 3.51	58.34 ± 4.17 ^a^
PSI baseline	<2	3.94
O’Leary PSI (%)	87.90 ± 9.33	88.82 ± 9.20 ^ns^
BOP (%)	55.67 ± 8.55	73.98 ± 8.14 ^a^
PPD (mm)	2.33 ± 0.59	4.56 ± 1.86 (min 2, max 10) ^b^
Clinical attachment loss (mm)	No loss	4.89 ± 2.09 (min 2, max 10) ^a^
Organoleptic score (0–4)	0 (No malodor)	3.29 ± 0.73 (2–4) ^a^

PSI: Periodontal Screening Index; PPD: pocket probing depth; BOP: bleeding on probing. Values are presented as mean ± SD. Statistical significance was analyzed using Student’s test: ^b^ *p* ˂ 0.01, ^a^ *p* ˂0.001, and ^ns^ not significant.

**Table 2:** Discriminant molecular features (MFs) acquired from samples from the halitosis with periodontitis group compared to the periodontally healthy group.

No.	Retention Time (min)	Regulation	*m*/*z*	Proposed Elemental Composition
1	5.3	Up	116.0714	C_5_H_9_NO_2_
2	5.5	Down	600.3079	Unidentified *
3	6.5	Up	203.1010	C_8_H_14_N_2_O_4_
4	7.4	H	517.2246	C_32_H_28_N_4_O_3_
5	7.8	C	139.0489	C_6_H_6_N_2_O_2_
6	9.2	Down	527.2835	C_28_H_38_N_4_O_6_
7	9.6	Down	600.2726	C_29_H_37_N_5_O_9_
8	11.9	Up	182.0798	C_9_H_11_NO_3_
9	12.2	H	132.1006	C_6_H_13_NO_2_
10	15.6	H	711.3402	C_38_H_50_N_2_O_11_
11	19.1	Down	367.1968	C_16_H_30_O_9_
12	20.3	Up	120.0821	C_8_H_9_N
13	21.4	Up	720.4030	Unidentified *
14	22.5	Up	216.0962	Unidentified *
15	22.7	C	155.0821	C_7_H_11_N_2_O_2_
16	24.5	Up	279.1317	C_14_H_18_N_2_O_4_
17	24.8	Up	458.2525	C_23_H_39_NO_6_S
18	27.9	Down	588.5022	C_37_H_65_NO_4_
19	28.4	H	188.0683	C_6_H_10_ClN_5_
20	29.9	Up	561.3016	Unidentified *
21	33.6	H	739.2892	Unidentified *
22	36.5	H	819.2567	Unidentified *
23	41.0	C	437.2343	C_28_H_28_N_4_O
24	41.9	Up	716.3843	C_25_H_50_N_17_O_6_S
25	42.8	C	525.2880	C_24_H_44_O_12_
26	45.3	Up	742.4410	Unidentified *

* No elemental composition predicted within molecular weight tolerance ± 10 ppm. H: present only in halitosis with periodontitis group; C: present only in control group.

## Data Availability

The datasets generated and utilized in this study are available from the corresponding author upon reasonable request.

## Supplementary Materials

The following supporting information can be downloaded at: https://www.mdpi.com/article/10.3390/dj13010036/s1, Figure S1: The microbial compositions of saliva samples from the halitosis with periodontitis and periodontally healthy groups based the relative abundance at the phylum level; Figure S2: The microbial compositions of saliva samples from the halitosis with periodontitis and periodontally healthy groups based on the relative abundance at the genus level; Figure S3: A representative GC-MS chromatogram from saliva samples analyzed using GC-MS; Table S1: Primers used for qPCR in this study; Table S2: Differentiating molecular features (MFs) between halitosis with periodontitis and periodontally healthy groups.
