# Supplementary material for: Relationship Between the Salivary Microbiome and Oral Malodor Metabolites in Older Thai Individuals with Periodontitis and the Cytotoxic Effects of Malodor Compounds on Human Oral Squamous Carcinoma (HSC-4) Cells
Source: Dent J (Basel). 2025 Jan 16;13(1):36. doi: 10.3390/dj13010036 (PMC11764442; doi:10.3390/dj13010036)
Supplement: Supplementary file 1 [file dentistry-13-00036-s001.zip › dentistry-3382564-supplementary.pdf]

Supplementary Information for

Relationship Between the Salivary Microbiome and Oral Malodor Metabolites in Older Thai Individuals with Periodontitis and the Cytotoxic Effects of Malodor Compounds on Human Oral Squamous Carcinoma (HSC-4) Cells

Witsanu Srila[1,2], Kritsana Sripilai[2], Thunwa Binlateh[4], Peungchaleoy Thammanichanon[3], Watcharaphol Tiskratok[3], Parinya Noisa[2] and Paiboon Jitprasertwong[3,*]

[1]Division of Biology, Faculty of Science and Technology, Rajamangala University of Technology Thanyaburi, Pathum Thani 12110, Thailand.

[2]Laboratory of Cell-Based Assays and Innovations, School of Biotechnology, Institute of Agricultural Technology, Suranaree University of Technology, Nakhon Ratchasima 30000, Thailand.

[3]Institute of Dentistry, Suranaree University of Technology, Nakhon Ratchasima 30000, Thailand.

[4]School of Pharmacy, Walailak University, Nakhon Si Thammarat 80160, Thailand.

* Corresponding author, email paiboonj@sut.ac.th

This file contains 3 supplementary figures and 2 supplementary tables.

Figure S1. The relative abundance of microbial compositions of saliva samples in the halitosis with periodontitis and periodontal health groups based on phylum level.

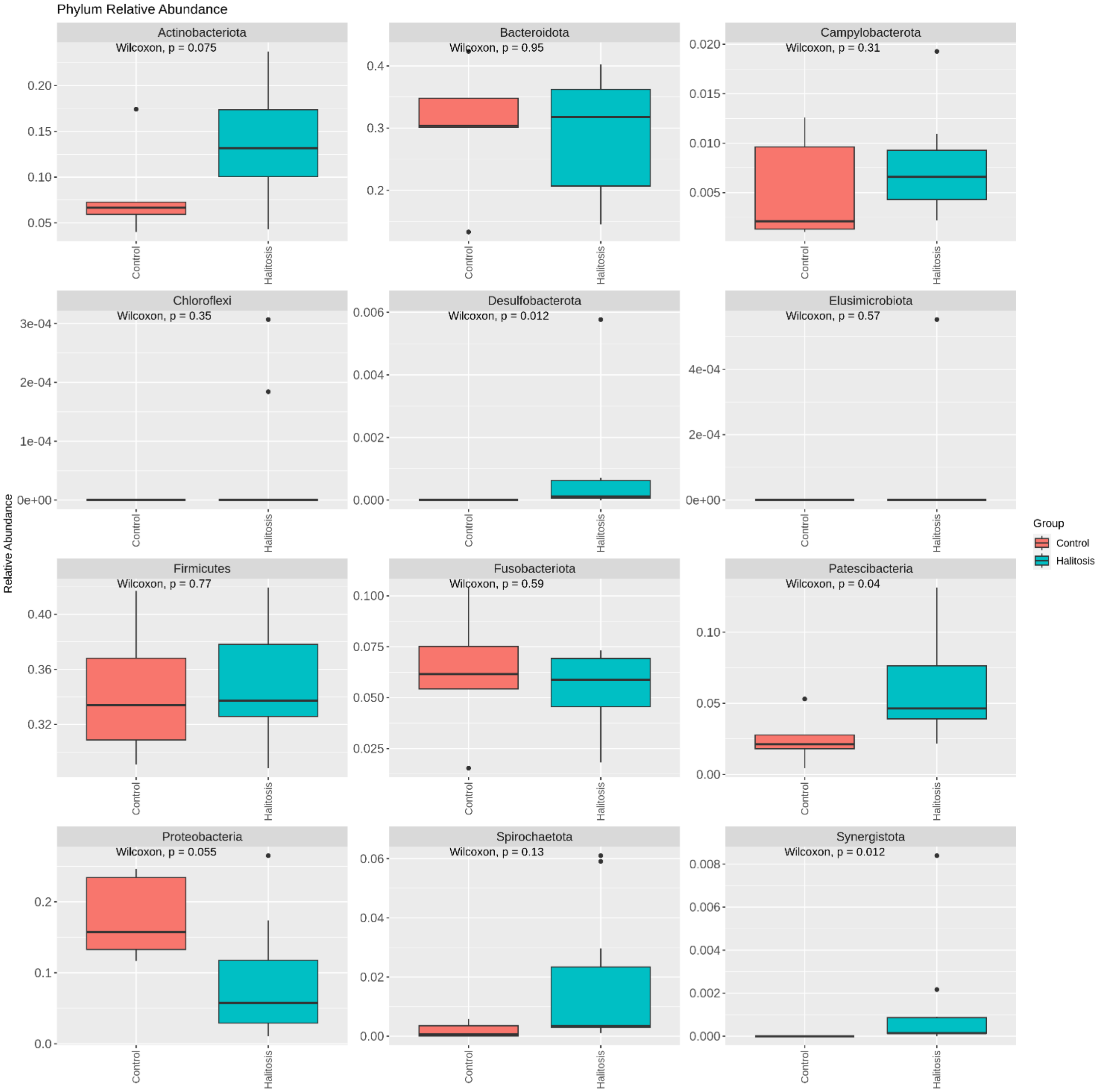

Figure S2. The relative abundance of microbial compositions of saliva samples in the halitosis with periodontitis and periodontal health groups based on genus level.

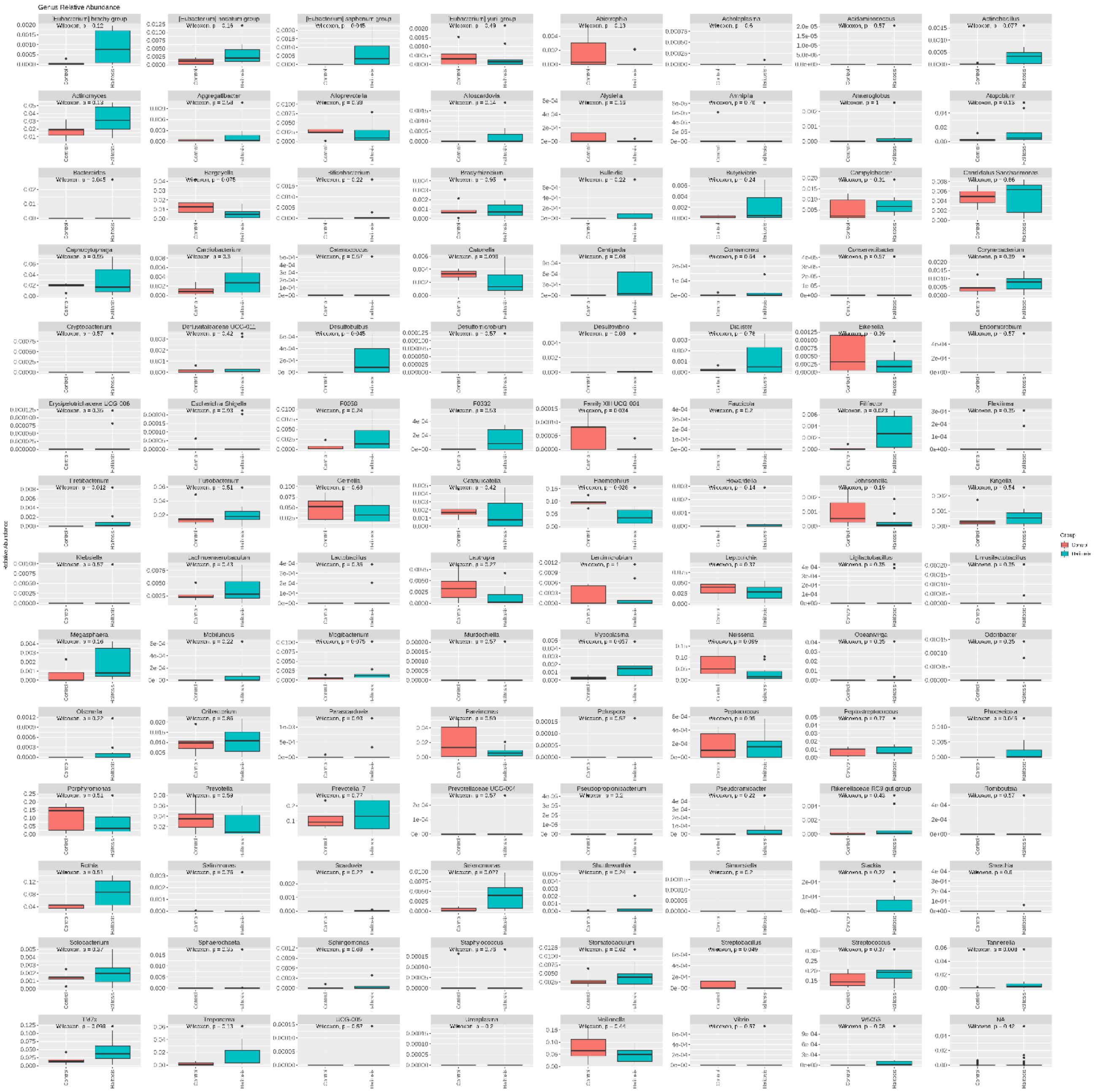

Breath Analysis by Headspace Solid-Phase Microextraction (HS-SPME) and Gas Chromatography Coupled to Mass Spectrometry (GC-MS) Method

Breath samples were collected in a 0.5 Tedlar® bag in the hospital. Then, the sample bags were executed to the laboratory and analyzed on the same day as sample collection by HS-SPME-GC/MS. VOCs were preconcentrated using a 65 μm polydimethylsiloxane (PDMS)/divinylbenzene (DVB) fiber (Agilent Technologies, USA). The fiber was incubated inside the bags at 37°C for 45 min. The gas-absorbed fiber was injected into a DB-Wax (60 m x 0.25 mmID x 0.25 μm film thickness) fused silica capillary column (J&W Scientific; Folsom, USA). The injector port was set at 240°C and split mode was applied with a split ratio of 20:1. Helium gas was used as the carrier with a constant flow rate of 1.0 mL/min. The GC oven temperature program was as follows: start at 40°C for 3 min, increase to 300°C at 10°C/min and hold for 5 min. Spectra acquisition was proceeded in electron ionization mode (at 70 eV) with the ion source temperature set at 250°C in scan mode and the mass scan range was from 30-500 *m*/*z*. The Agilent MassHunter Qualitative Analysis B.04.00 software was used for data analysis. Identification of VOCs was done by comparing their mass spectra with NIST mass spectral libraries (National Institute of Standards, 2011 version). The VOC content was calculated based on the peak area.

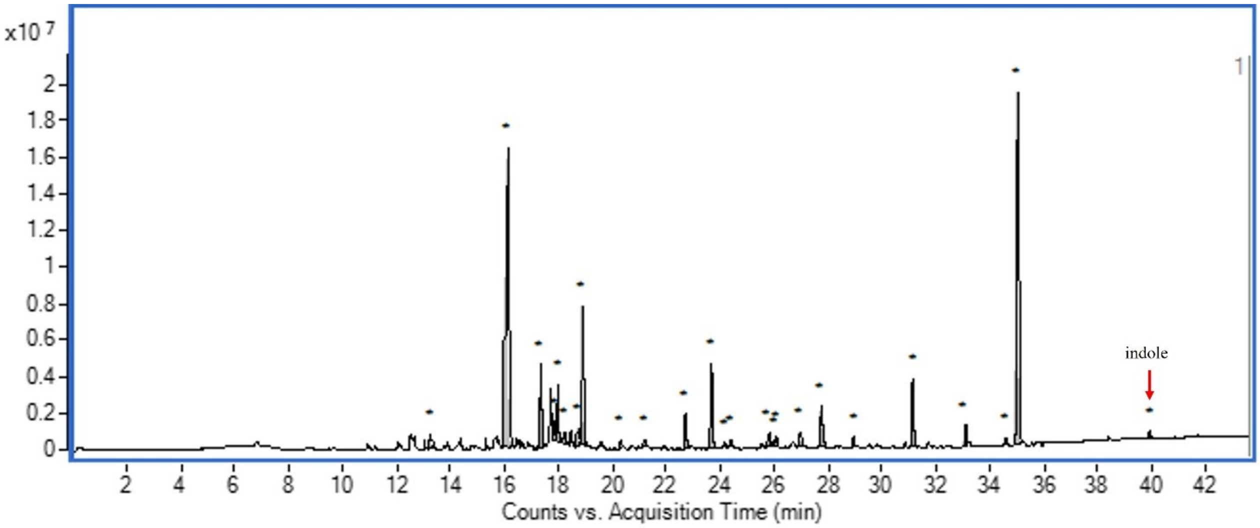

Figure S3. A Representative of GC-MS chromatogram from saliva samples analyzed by GC-MS.

Table S1. Primer used for qPCR in this study.

Name	Forward (5'-3')	Reverse (5'-3')	Gene-related study
GAPDH	ACCTGACCTGCCGTCTAGAA	TCCACCACCCTGTTGCTGTA	House keep
SOD	CTAGCGAGTTATGGCGAC	CATTGCCCAAGTCTCCAAC	Antioxidant
CAT	TCCGGGATCTTTTTAACGCCATTG	TCGAGCACGGTAGGGACAGTTCAC	
GPX	CGCCAAGAACGAAGAGATTC	CAACATCGTTGCGACACAC	
BAX	CCTTTTCTACTTTGCCAGCAAAC	GAGGCCGTCCCAACCAC	Apoptosis
CAS8	CTCCCCAAACTTGCTTTATG	AAGACCCCAGAGCATTGTTA	
CAS3	GCTTGTCGGCATACTGTTTCAG	AGAACTGGACTGTGGCATTGAG	
IL6	TCTCCACAAGCGCCTTCG	CTCAGGGCTGAGATGCCG	Inflammatory
TNF-α	GAGCACTGAAAGCATGATCC	CGAGAAGATGATCTGACTGCC	

Table S2. Differentiating molecular features (MFs) between halitosis with periodontitis and periodontal health groups.

No.	Retention time (min)	Measured mass	Accurate mass	Regulation (Fold)	Proposed elemental composition	Tentative annotation
1	5.3	116.0714	116.0706	Up (1.90)	$C_5H_9NO_2$	N-Allylglycine, 5-Hydroxy-1-methylpyrrolidin-2-one, N-(2-Hydroxyethyl)acrylamide ,D/L-Proline
2	5.5	600.3079	-	Down (0.82)	Unidentified	-
3	6.5	203.1010	203.1026	Up (2.58)	$C_8H_{14}N_2O_4$	Prolyl-Serine, Hydroxyprolyl-Alanine, L-Coprine
4	7.4	517.2246	517.2234	H	$C_{32}H_{28}N_4O_3$	1-(3-Methylphenyl)-3-[(3R)-1-[2-(2-methylphenyl)-2-oxoethyl]-2-oxo-5-phenyl-3H-1,4-benzodiazepin-3-yl]urea
5	7.8	139.0489	139.0502	C	$C_6H_6N_2O_2$	6-Hydroxynicotinamide,5-Methylpyrazine-2-carboxylic Acid, 3-Aminopicolinic acid, 3-Nitroaniline
6	9.2	527.2835	527.2864	Down (0.48)	$C_{28}H_{38}N_4O_6$	Val-Phe-Val-Tyr
7	9.6	600.2726	600.2664	Down (0.44)	$C_{29}H_{37}N_5O_9$	Gluten exorphin A5
8	11.9	182.0798	182.0812	Up (1.70)	$C_9H_{11}NO_3$	2-Amino-3-hydroxy-3-phenylpropanoic acid, 4,6,7-Trihydroxy-1,2,3,4-tetrahydroisoquinoline, D-Tyrosine
9	12.2	132.1006	132.1019	H	$C_6H_{13}NO_2$	Methyl valine, 3-Amino-4-methylpentanoic acid, beta-alanine betaine, D/L-Norleucine
10	15.6	711.3402	711.3487	H	$C_{38}H_{50}N_2O_{11}$	Andersoline
11	19.1	367.1968	367.1963	Down (0.35)	$C_{16}H_{30}O_9$	(2R,6x)-7-Methyl-3-methylene-1,2,6,7-octanetetrol 2-glucoside
12	20.3	120.0821	120.0808	Up (4.01)	C_8H_9N	Isoindoline, Indoline (2,3-dihydro-1H-indole)
13	21.4	720.4030	-	Up (1.30)	Unidentified	-
14	22.5	216.0962	-	Up (2.35)	Unidentified	-
15	22.7	155.0821	155.0815	C	$C_7H_{11}N_2O_2$	6-n-Propyluracil, N,N'-Methylenebisacrylamide, Hexahydropyrrolo[1,2-a]pyrazine-1,4-dione

Table S2. Differentiating molecular features (MFs) between halitosis with periodontitis and periodontal health groups. (continue)

No.	Retention time (min)	Measured mass	Accurate mass	Regulation (Fold)	Proposed elemental composition	Tentative annotation
16	24.5	279.1317	279.1339	Up (2.54)	$C_{14}H_{18}N_2O_4$	4'-Hydroxymethohexital, Musk moskene, Tyrosyl-Proline, N1-(alpha-D-ribosyl)-5,6-dimethyl-benzimidazole
17	24.8	458.2525	458.2571	Up (9.2)	$C_{23}H_{39}NO_6S$	10,11-Dihydro-12R-hydroxy-leukotriene E4
18	27.9	588.5022	588.4986	Down (0.27)	$C_{37}H_{65}NO_4$	Cer(d17:1/20:4(5Z,7E,11Z,14Z)-OH(9))
19	28.4	188.0683	188.0697	H	$C_6H_{10}ClN_5$	6-Chloro-N-(1-methylethyl)-1,3,5-triazine-2,4-diamine
20	29.9	561.3016	-	Up (1.26)	Unidentified	
21	33.6	739.2892	-	H	Unidentified	
22	36.5	819.2567	-	H	Unidentified	
23	41.0	437.2343	437.2336	C	$C_{28}H_{28}N_4O$	Depropionylbezitramide
24	41.9	716.3843	-	Up (2.29)	$C_{25}H_{50}N_{17}O_6S$	
25	42.8	525.2880	525.2906	C	$C_{24}H_{44}O_{12}$	Sucrose monolaurate
26	45.3	742.4410	-	Up (1.21)	Unidentified	
